# A discrete event simulation model to evaluate the use of community services in the treatment of patients with Parkinson’s disease in the United Kingdom

**DOI:** 10.1186/s12913-017-1994-9

**Published:** 2017-01-18

**Authors:** Reda Lebcir, Eren Demir, Raheelah Ahmad, Christos Vasilakis, David Southern

**Affiliations:** 1 University of Hertfordshire, AL10 9AB, Hatfield, UK; 2 Faculty of Medicine, Imperial College London, Hammersmith Campus, du Cane Road, London, W12 0NN UK; 3 University of Bath, BA2 7AY, Bath, UK; 4 Pathways Communications, CB8 8RW, Kennett, UK; 5 Health Group, Management Department, Imperial College Business School, Exhibition Road, London, SW7 2AZ UK

**Keywords:** Parkinson’s disease, Community services, Discrete event simulation, National health service, United Kingdom

## Abstract

**Background:**

The number of people affected by Parkinson’s disease (PD) is increasing in the United Kingdom driven by population ageing. The treatment of the disease is complex, resource intensive and currently there is no known cure to PD. The National Health Service (NHS), the public organisation delivering healthcare in the UK, is under financial pressures. There is a need to find innovative ways to improve the operational and financial performance of treating PD patients. The use of community services is a new and promising way of providing treatment and care to PD patients at reduced cost than hospital care. The aim of this study is to evaluate the potential operational and financial benefits, which could be achieved through increased integration of community services in the delivery of treatment and care to PD patients in the UK without compromising care quality.

**Methods:**

A Discrete Event Simulation model was developed to represent the PD care structure including patients’ pathways, treatment modes, and the mix of resources required to treat PD patients. The model was parametrised with data from a large NHS Trust in the UK and validated using information from the same trust. Four possible scenarios involving increased use of community services were simulated on the model.

**Results:**

Shifting more patients with PD from hospital treatment to community services will reduce the number of visits of PD patients to hospitals by about 25% and the number of PD doctors and nurses required to treat these patients by around 32%. Hospital based treatment costs overall should decrease by 26% leading to overall savings of 10% in the total cost of treating PD patients.

**Conclusions:**

The simulation model was useful in predicting the effects of increased use of community services on the performance of PD care delivery. Treatment policies need to reflect upon and formalise the use of community services and integrate these better in PD care. The advantages of community services need to be effectively shared with PD patients and carers to help inform management choices and care plans.

**Electronic supplementary material:**

The online version of this article (doi:10.1186/s12913-017-1994-9) contains supplementary material, which is available to authorized users.

## Background

Neurodegenerative conditions and motor neurone affecting cognitive and physical ability have life changing impact on individuals, families and carers. Medical interventions are of limited benefit for these progressive illnesses if social care and support for managing daily activities is lacking. Fundamental to the management of such conditions is the coordination of care across different health and social care sectors. From a health systems perspective, models of integrated and efficient service delivery and care are required particularly in the context of aging populations; a challenge particularly in high income countries. Impact extends across the healthcare economy (primary, community, secondary and tertiary care) as well as to other public services including transport, employment and housing needs [1]. For long-term progressive conditions, key questions arising are *which treatment pathway?* and *where?* to achieve the best outcome for patients and healthcare providers and carers. To answer these questions we focus on one particular condition, Parkinson’s disease (PD) in a country where the population age profile is changing, the United Kingdom (UK).

PD is the second common chronic neurodegenerative condition in older people especially beyond the age of sixty [2, 3]. The most common early symptoms of the disease are problems with movement and this includes tremor, stiffness, slowness, and paucity of movement [4]. As the disease progresses, additional non-motor symptoms such as depression, psychotic symptoms, dementia, sleep disturbance, falls, and autonomic disturbances become more common adding to the burden of the disease and its effects on patients and those involved in their treatment and care. The late phases of the disease are generally characterised by increased motor complications, disability, and mortality [5].

PD is a challenging disease from a clinical perspective as it affects older adults who may also be suffering from other conditions. There is difficulty in diagnosis especially in early stages, the condition is associated with a wide range of symptoms, is progressive and there is currently no cure for the disease. As a result, the treatment and management of PD is complex and resource intensive due to the involvement of a multidisciplinary team and several services in its management. The complexity is increased by the fact that the disease does not just affect the patient, but the effects extend to families, employers, and a wide range of public services [1, 5].

The disease progresses over four phases and these impact directly on the treatment provided to patients [6]. The Diagnosis phase reflects the initial stage when a patient is suffering from PD with symptoms and signs, but there is no formal confirmation that the patient has PD. Once diagnostic tests are conducted and PD is confirmed, the patient moves to the Maintenance phase. Depending on the patient status, either no treatment is provided at this phase or treatment involving small doses of one or two drugs is commenced. As there is no cure for PD, the aim of the treatment is to slow the progression of the disease so that patient quality of life is not affected. As the symptoms worsen and more functions of the body are affected, the patient enters the Complex phase in which the number of drugs and their doses are increased and, in some cases, neurosurgery is performed. The final phase, known as Palliative, is characterised by high risks of physical and mental disabilities and significant threats to life.

The treatment modes for PD include inpatient, outpatient, and community care and require a range of clinical specialists [7]. Inpatient care involves drug treatment and surgery in hospitals. Outpatient care provides drug treatment and, in some instances, specialised therapies such as Physiotherapy, Psychiatry, Occupational Therapy, and Speech and Language Therapy. This care takes place mostly in General Practitioners (GPs) clinics and sometimes in hospitals. Community care, which focuses on specialised therapies, is provided in local care units, under Community Services, with the support of local pharmacies and social services.

The UK population is aging and, therefore, it is expected that the number of people who will be diagnosed with PD will increase in the future. In 2012, the number of people aged 65 years and over was 10.84 million from which 1.44 million were aged 85 years and over. The number of people aged 65 years and over is expected to rise to 17.79 million from which 3.64 million will be aged 85 years and over in 2037 [8]. The latest available data suggest that the number of individuals living with PD in the UK is 127,000, corresponding to a prevalence rate of 27.4/10,000 [9]. This number is expected to rise in line with the population ageing trend and to reach 165,000 in 2020 [9].

Healthcare in the UK is delivered free of charge, at the point of care, by a public organisation known as the “National Health Service (NHS)”. Current evidence suggests that the NHS is struggling to cope with the demand for PD treatment and care. A National Audit Office report found that although access for patients with neurological conditions including PD to health services improved since 2007, spending increased by 38% in real terms and the quality of care declined during the same period [1]. Just over half (66%) of GP referrals meet the National Institute for Health and Care Excellence (NICE) guidelines of a specialist appointment within 6 weeks and 14% of patients are readmitted within 28 days of discharge. Recent work has shown that less than a quarter of PD patients (22%) have a personal care plan [10].

This situation is alarming especially as the NHS is faced with additional pressures stemming from ever increasing resource and capacity constraints and the need to improve operational and cost efficiency. The NHS has a target of making a net saving of £20 Billion over the coming 4–5 years [11]. However, given its sheer size and complexity (the NHS employs more than 1.6 million people and deals with more than 1 million patients every 36 h [12]), the challenges are significant.

In this new reality of “doing more with less”, it is necessary to identify areas where efficiency gains can be made. This is challenging in the case of PD given the wide range of treatment modes and therapies required by patients, the different settings where the treatment is provided, and the need to customize the treatment at an individual patient level depending on their disease stage, severity of the symptoms and support structures available. For example, some patients may be seen by a PD doctor or by a PD nurse monthly, quarterly or every 6 months, for single or multiple therapies. A better understanding of the different possible configurations to deliver treatment and care to PD patients and the evaluation of the operational and cost performance of these configurations is required so that the most efficient ones are identified.

Treatment configurations involving increased use of community services could yield significant efficiency gains. Treating more patients in community services may reduce unnecessary hospital admissions, the need for consultations with PD doctors and nurses, and facilitate the earlier discharge if admitted [5]. Treating patients in community services has a significant cost advantage over treatment in hospitals. The average cost to treat a PD patient admitted to inpatient care as an emergency admission is £2,133 (based on an average length of stay of 6.3 days) and the average cost of a PD doctor visit is around £145. In contrast, the costs associated with community services are in the region of £38 to £98 (e.g. physiotherapy £38, occupational therapy £56 and speech and language therapy £98) [13]. The additional benefit is that community services improve patients’ understanding of their own disease journey and empower them to better self-manage their condition. This is why they have been welcomed by patients [5].

The lack of research to evaluate operational and cost performance of possible configurations is surprising given the potential efficiency gains which could be achieved through increased deployment of community services. The vast majority of research, with regard to PD performance evaluation, has focused on quality of life of patients and the effects of PD symptoms and social factors on the physical and psychological wellbeing of patients [14–17]. The literature covering the evaluation of PD care configurations includes only a 2011 report by the UK National Audit Office, which recommends the use of community services and recently published research providing some evidence that the use of community services reduces PD doctors and nurses’ activity levels [7]. This is a clear research gap, which warrants further investigation.

The aims of this study are therefore: First, to develop a Discrete Event Simulation (DES) model, which captures the PD treatment pathways and service configurations including community services; Second, to determine possible and realistic policies, which could be implemented with regard to an increased use of community services and how these would affect hospital based treatment; Third, to evaluate the impact of the implementation of these policies on a number of operational and cost performance indicators relevant to the delivery of PD treatment and care. This should provide an indication of the feasibility and scale of efficiency improvement, which could be achieved by shifting more PD patients to community services and allow health managers to make informed decisions with regard to the best ways to reconfigure the PD care delivery system in the UK.

## Methods

We developed a Discrete Event Simulation (DES) model [18–20] to portray the structure and configuration of the PD treatment and care system including patients’ pathways, disease phases, treatment modes, and the resources required to provide treatment and care. DES is appropriate in the context of this research as it enables the modelling of patients at the individual level including disease evolution over time and how a patient moves through the different parts of the care system. DES makes it possible to represent different patient general attributes such as age and gender and clinical specific attributes such as disease phase and treatment mode. As time progresses, patients’ attributes are altered to reflect changes in their status. This allows the tracking of patients as they evolve in the care system including the events they experience at different points of time. DES provides the flexibility to incorporate capacity and resource constraints explicitly, and to capture the resource allocation policies and priority rules where entities compete for limited resources. This feature is extremely important in health contexts, as clinical activities such as diagnosis, treatment, and consultations require a mix of specialised resources (doctors, nurses, beds, operating theatres, and so on) and these resources are, in most cases, not sufficient to meet the required level of demand.

DES has been applied to health management since the 1990s driven by the increased complexity of health care systems, the considerable advancements in DES software capabilities, ease of use, and the shift to more evidence based decision making in the health sector [21–24]. The increased popularity of DES in health management is reflected in the literature, which show an upward trend in terms of the number of DES applications and the areas covered by these applications [23, 25]. Examples of these applications include modelling of patient flows in hospitals and accident and emergency departments in particular [26, 27], reconfiguration of care delivery services and capacity planning and management in the health sector [28–31], cost effectiveness of treatment procedures [32–35], patients’ compliance with screening procedures [36], and policies to prevent transmission of diseases [37].

### Simulation Model Development

The model was developed in collaboration with health care professionals and potential end-users and its building process started with a meeting with the National Committee of the Parkinson’s Disease Nurse Specialist Association (PDNSA) in late 2012. Six PD specialist nurses were involved and provided detailed information regarding patients’ pathways and treatment procedures, the services and resources required in the diagnosis and treatment of PD, and the rules and policies associated with the management of the patients. Semi structured interviews were conducted with the nurses and led to an initial mapping of the PD patients’ pathway structure. This map was further developed and refined through structured interviews with members of the PDSNA national committee, who checked that the initial map reflected the patients’ pathways and suggested corrections where gaps were identified.

**Fig. 1:**
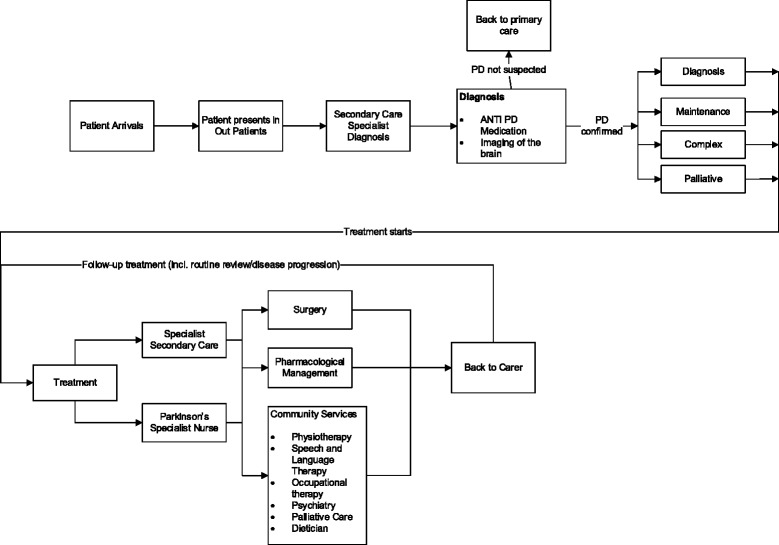
Structure of the Parkinson’s disease patients’ pathway. Adapted from: Demir et al. 2015 [7]

The interviews indicated that the first point of contact of a patient with the PD care system is a visit to a PD outpatient clinic following referral from a General Practitioner (GP), Accident and Emergency (A&E) units, outpatient department, or a hospital department. Diagnostic tests are carried out, and, if PD is confirmed, patients are categorised into one of the disease phases (Diagnosis, Maintenance, Complex or Palliative) and treatment commences. The treatment is supervised by a PD doctor and takes the form of a referral to surgery (in a small number of cases), treatment via drugs, special therapies (eg Physiotherapy, Language and Speech Therapy, Occupational Therapy) in community services, or a combination of these treatment modes (for example treatment via drugs and special therapies) (See Fig. 1) The PD specialist overseeing the treatment of each individual patient is supported by a PD specialist nurse, who plays a critical role in the management of the PD patient and determination of their treatment needs.

As there is no known cure for PD, the treatment is a continuous process over time and the patient remains in treatment indefinitely. Patients meet their allocated PD doctor and PD nurse on a regular basis and the frequency of these meetings depends on the disease phase of the patient. The treatment guidelines indicate that patients in the Diagnosis, Maintenance, Complex and Palliative phases meet with the PD doctor and PD nurse 2, 4, 5 and 6 times a year respectively. In these meetings, disease progression is evaluated and decisions made about changes to drug combinations. Requirements regarding the use of primary care or community services for special therapies are also determined in these meetings depending on the medical state of the patient.

One important aspect of the meetings between the PD doctor and the patient is the assessment of the medical status of the patient and the decision to keep or change the disease phase of the patient. As described earlier, once a patient is formally diagnosed with PD, that patient is allocated to one of the four phases of the disease (Diagnosis, Diagnosis, Maintenance, Complex, Palliative) as this determines the treatment process and the frequency of the patient’s meetings with the PD doctor and PD nurse. The decision to keep or alter the patient’s phase will, consequently, have important implications for the treatment and management of patients as it affects the level of resources required for the treatment and the needs in terms of the special therapies prescribed for the patient.

**Fig. 2:**
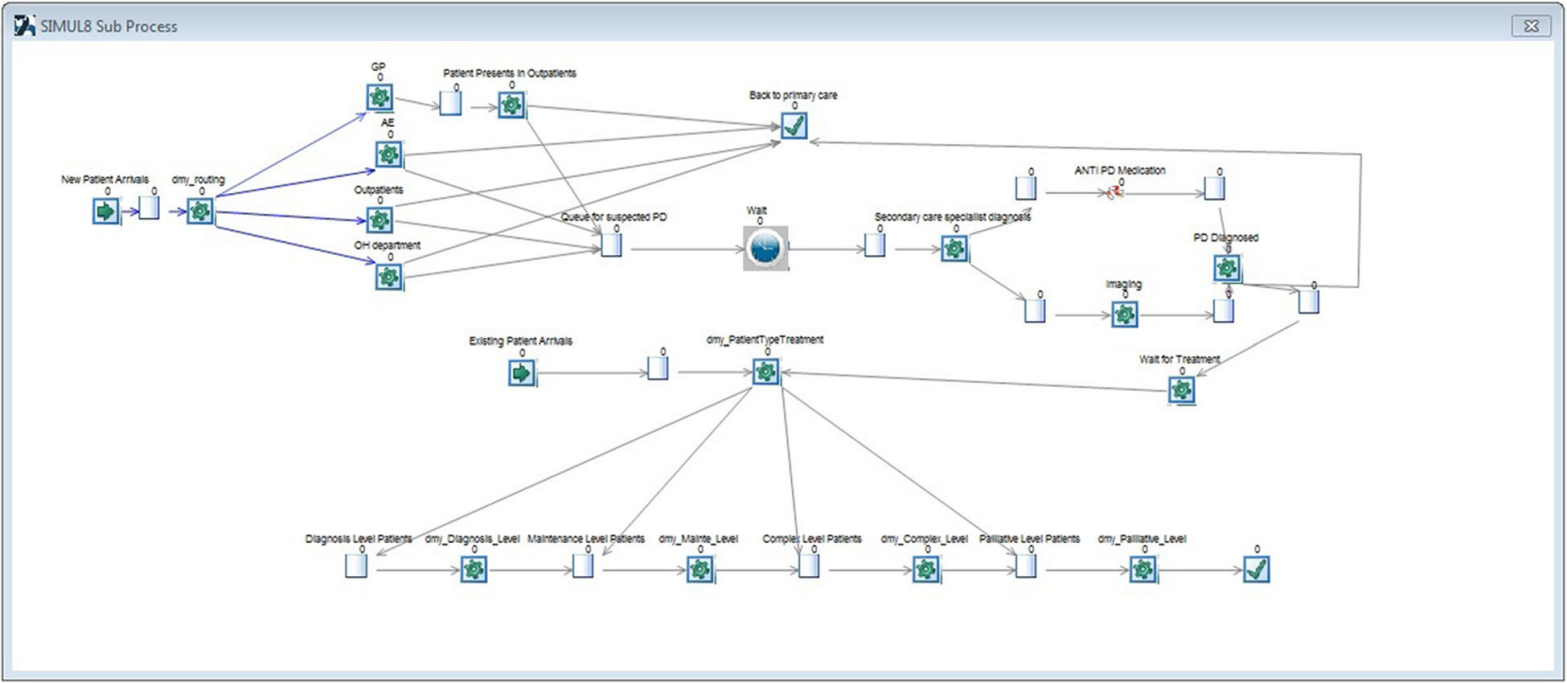
SIMUL8 snapshot of the diagnosis process for Parkinson’s disease patients

The information generated in the interviews regarding the PD patients’ pathways (shown in Fig. 1) and how the patients are treated and managed were entered in a simulation model built using the software SIMUL8 (www.simul8.com). The model represented the four different sources (GPs, A&E units, outpatient department, hospital department), which bring patients to the PD care system, progression through different disease phases (Diagnosis, Maintenance, Complex, and Palliative), treatment modes, the mix of resources required for the treatment, and the special therapies provided in community services (see Fig. 2 for a snapshot of the SIMUL8 model).

**Fig. 3:**
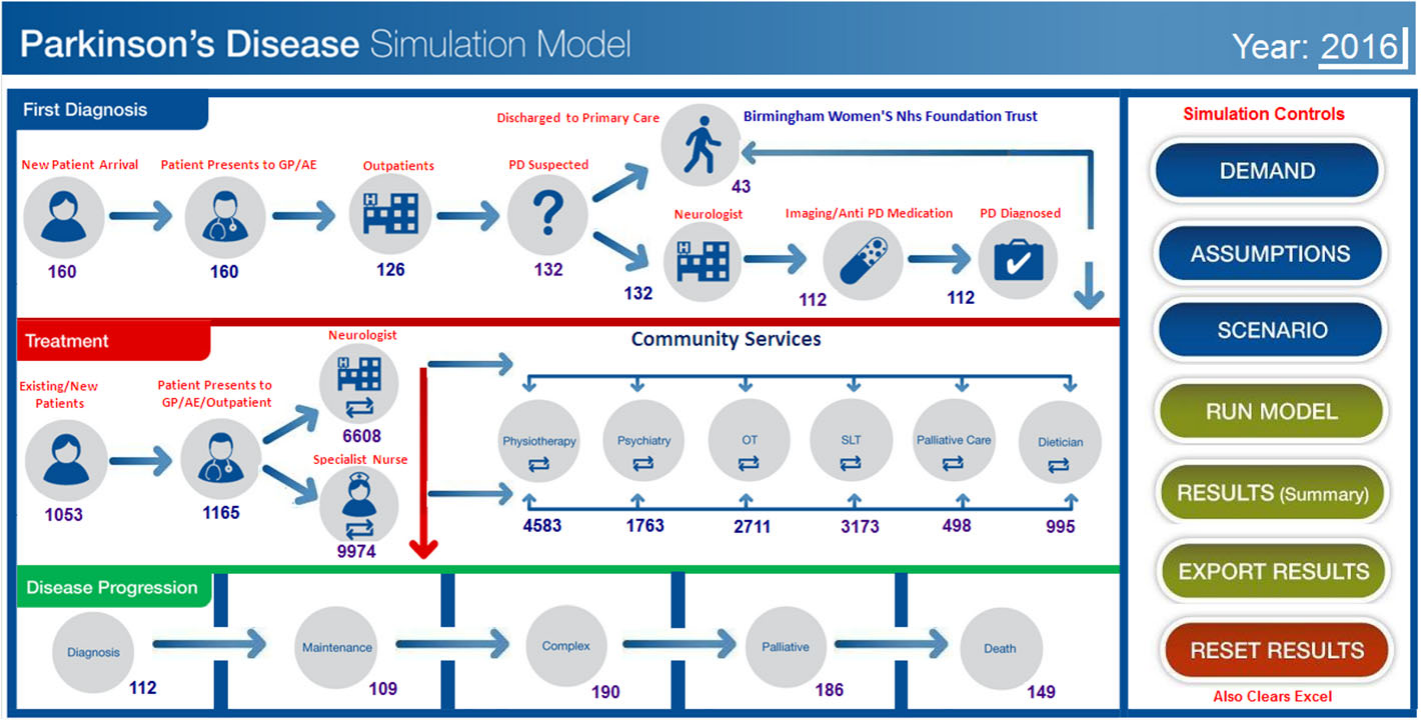
The Parkinson’s disease simulation model user interface

As the model is aimed for decision makers, a friendly and easy to use interface was added to the SIMUL8 model. The interface is animated and allows users to test policies regarding changes to demand levels, patient pathways, allocation of resources, and disease progression (See Fig. 3). These policies are represented in the form of scenarios, which the decision makers could input on the interface and then run the model. Once a scenario run is completed, the model generates a results summary on the key performance indicators of interest to decision makers, which are also shown on the model interface. In addition, the model generates a complete set of results which can be exported to an Excel spreadsheet to enable more detailed analysis.

### Model parameters and validation

Several sources of information were used to estimate the values of the parameters entered in the model (see Additional file 1: Table S7). The NHS English Hospital Episodes Statistics (HES) data set (the biggest and most comprehensive official health statistics database in the UK) and interviews with PD doctors and nurses were the sources for parameters regarding origin of referrals, PD progression and treatment, and provision of community services.

Given that PD treatment in the UK is decentralised and entities, known as NHS Trusts, are responsible for providing care at a regional level, the parameters related to the number of patients and treatment modes were estimated from one big NHS Trust, which provides a full set of treatment modes including special therapies in community services. In this Trust, the breakdown of new PD patients by source of referral is 75%, 15%, 5%, and 5% for GP, A&E, outpatients, and other hospital department respectively. The distribution of patients by disease stage category is mostly constant at 10% Diagnosis, 60% Maintenance, 25% Complex and 5% Palliative. Visits to PD doctors occur once a year in the Diagnosis phase, 2 to 3 times a year in the Maintenance phase, and 3 to 4 times a year in the Complex and Palliative phases. Patients are seen 2 to 4 times a year by a PD nurse except in the Palliative stage where a customised care plan is designed and patients are seen by a PD nurse once a month.

Information regarding the use of community services is scarce and mostly unrecorded, so the research team relied on the extensive experience of PD nurses to estimate the parameters related to these services. Following interviews, we determined the percentage of patients referred to community services including those using a single specialised therapy (eg physiotherapy) or a combination of therapies (eg speech and language therapy and occupational therapy). The number of visits per year to community services including visits to single or combination of specialised therapies and the community services therapy specialists required in these visits were also determined from the interviews. The breakdown of patients using the different types of therapies in the Trust from which the data was collected was confirmed by senior PD doctors and nurses to be, on average, equal to 45% for physiotherapy, 42.5% for speech and language therapy, 35% for occupational therapy, 22.5% for psychiatry services, 7.5% for dietician, and 7.5% for palliative care.

The unit costs associated with the different modes of treatment (hospital, outpatient, community services) were determined using the Healthcare Resource Group (HRG) code, which provide standardised and reference costs for the treatment of different diseases and clinical conditions in the UK. The HRG codes and their associated costs are publicly available on the UK Department of Health website [13]. These codes enabled us to estimate the treatment costs for the different treatment modes including those related to the provision of specialised therapies in community services. Extra costs were included to reflect instances of unexpectedly long stay in hospitals or additional treatments and tests. The number of staff available and their salaries were determined from interviews. The list of data entered in the model is presented is included in an additional file with this paper.

The model was put to validation tests to ensure that it can be confidently used to simulate alternative scenarios regarding the treatment procedures of PD patients and determine the most appropriate policies out of the simulation results. The validation tests covered two aspects: (i) the model’s ability to replicate historical observations and (ii) the extent to which the model was a correct representation of the PD care system (face validity). Data representing the current situation in the selected Trust was entered into the model and then run for a period of 3 years and the results generated by the model were compared to those taken from the Trust data. The model results were very close to those observed in the real world on a number of variables for which real world data were available. A summary of the simulation and real world results for this validation test are presented in Table 1.

**Table 1:** Simulation and real world results for model validation

Performance Indicator	Simulation Results Mean 95% (LCI, UCI)	Real World Results	Difference (in Percentage)
PD doctors’ visits	8225 (7732, 8801)	8061	2% (−4%, 8%)
PD nurses visits	10357 (10046,10771)	10875	−5% (−8%, −1%)
PD doctors service hours	8225 (7978, 8554)	8061	2% (−1%, 5%)
PD nurses service hours	10357 (10150, 10978)	10875	−5% (−7%, 1%)
PD doctors total FTEs	1.07 (0.995, 1.123)	1.04	3% (−4%, 7%)
PD nurses total FTEs	3.4 (3.230, 3.502)	3.57	−5% (−10%, −2%)

Face validity was performed by showing the PD care system map and the simulation model to each nurse individually and then to all nurses in a group workshop. The model structure was confirmed to be highly representative of the real world PD care system by all nurses. The engagement of the nurses and their continuous involvement and feedback was instrumental in achieving face validity for the model.

## Results

In addition to building the DES model, this research has two additional aims, which are (i) to determine the possible and realistic policies, which could be implemented with regard to an increased use of community services in the treatment of PD patients and (ii) to evaluate the impact of the implementation of these policies on a number of operational and cost performance indicators relevant to the delivery of PD treatment and care. Following extensive discussions with decision makers and experienced PD doctors and nurses, four possible scenarios were determined and selected for simulation on the model. These scenarios are as follows:

- *Scenario 1*: *Low increase in community services*. Under this scenario, 10% more patients will be shifted to treatment in community services.
- *Scenario 2*: *Medium increase in community services*. Under this scenario, 20% more patients will be shifted to treatment in community services.
- *Scenario 3*: *High increase in community services*. Under this scenario, 40% more patients will be shifted to treatment in community services.
- *Scenario 4*: *Very high increase in community services*. Under this scenario, 50% more patients will be shifted to treatment in community services.

Following the selection of scenarios, further discussions were carried out with the decision makers to determine the most appropriate indicators, which could be used to evaluate the operational and cost performance of the different PD treatment configurations reflected by the scenarios. These indicators were divided into three categories: (i) Level of Activity (ii) Resources Requirements and (iii) Cost of Care. The full list of performance indicators is presented in Table 2.

**Table 2:** Definition of the performance indicators

Performance Indicator	Definition
Level of Activity	
PD doctors’ visits	Number of consultations of PD patients with doctors in hospitals
PD nurses visits	Number of consultations of PD patients with nurses in hospitals
Physiotherapy visits	Number of consultations to provide Physiotherapy to PD patients
SLT therapy visits	Number of consultations to provide SLT therapy to PD patients
Psychiatry visits	Number of consultations to provide Psychiatry therapy to PD patients
Occupational therapy visits	Number of consultations to provide Occupational therapy to PD patients
Palliative care visits	Number of consultations to provide Palliative care therapy to PD patients
Dietician visits	Number of consultations to provide Dietician therapy to PD patients
Resource Requirements	
PD Doctors service hours	Number of PD doctors working hours required to provide treatment to patients
PD nurses service hours	Number of PD nurses working hours required to provide treatment to patients
PD Doctors Total FTEs	Number of Full Time Equivalent (FTEs) of PD doctors required to provide treatment to patients
PD Nurses Total FTEs	Number of Full Time Equivalent (FTEs) of PD nurses required to provide treatment to patients
Treatment Costs	
PD Doctors cost	Total PD doctors costs required to treat patients in hospitals
PD Nurses cost	Total PD nurses costs required to treat patients in hospitals
Physiotherapy costs	Total costs required to provide Physiotherapy to PD patients
SLT costs	Total costs required to provide SLT to PD patients
Psychiatry costs	Total costs required to provide Psychiatry therapy to PD patients
Occupational therapy costs	Total costs required to provide Occupational therapy to PD patients
Palliative care costs	Total costs required to provide Palliative care therapy to PD patients
Dietician costs	Total costs required to provide Dietician therapy to PD patients

To achieve robustness of the results, each scenario was run 100 times (using different random numbers each time) for a period of three years with a “warm up” period of 1 year. Results were collected on the performance indicators for the four scenarios. An analysis of these results suggests the following

**Fig. 4:**
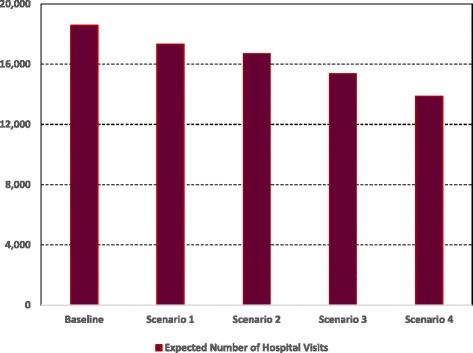
Expected number of hospital visits

**Fig. 5:**
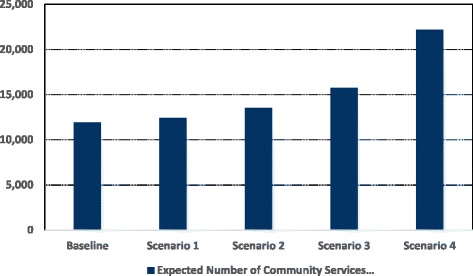
Expected number of community services visits
- *Resource Requirements*: The level of hospital resources, expressed in terms of the total number of Full Time Equivalent (FTEs) for hospital doctors and nurses, required to treat PD patients should go down as more patients are treated in community services rather than in hospitals (See Table 4). The decrease in the number of required FTEs becomes more important as higher fractions of patients are directed to community services. Specifically, the percentage reduction in FTEs will be 7% and 9% under scenarios 1 and 2 respectively before increasing to 16% under scenario 3 and then doubling to 32% under scenario 4.

- *Level of Activity*: The results indicate that the policy to shift more treatment activities to community services will significantly ease the workload of hospital based staff (See Table 3). The expected number of visits to hospitals decreases steadily from 18582 visits under the baseline scenario to 13889 visits under scenario 4. This trend becomes steeper as the fraction of patients treated in community services goes up (See Fig. 4). Starting from the baseline scenario, the percentage decline in the expected number of visits to hospitals is 7%, 10%, and 17% under scenarios 1, 2, and 3 respectively and reaches a maximum value of 25% under scenario 4.The impact of the policy on the number of visits to community services therapies is much more important. The expected number of these visits should increase slightly under scenario 1 (by approximately 4% from 11910 to 12397 visits) and then the rate of increase will become more significant at 14% and 33% under scenarios 2 and 3 respectively. However, for scenario 4, the increase in the expected number of visits becomes extremely sharp with a total of 22211 visits, which is equivalent to an 86% increase compared to the baseline scenario (See Fig. 5).

**Table 3:** Average, 95% lower and upper CI results for the number of visits to PD doctors and nurses

Performance Indicator	Baseline	Scenario 1: Low use of CS	Scenario 2: Medium Use of CS	Scenario 3: High Use of CS	Scenario 4: Very High Use of CS
PD doctors’ visits	8225 (7732, 8801)	7494 (7194, 7944)	7158 (6872, 7516)	6537 (6145, 6798)	5873 (5638, 6108)
PD nurses visits	10357 (10046,10771)	9854 (9263,10248)	9553 (8980, 10126)	8831 (8301, 9449)	8016 (7695, 8417)
Total Hospital Visits	18582 (17778, 19572)	17348 (16457, 18192)	16711 (15852, 17642)	15368 (14446, 16247)	13889 (13333, 14525)
Physiotherapy visits	3554 (3341, 3767)	3700 (3552, 3922)	4036 (3915, 4319)	4708 (4567, 5038)	6755 (6552, 7228)
SLT visits	3357 (3256, 3592)	3494 (3389, 3704)	3811 (3620, 4002)	4447 (4314, 4714)	6379 (6124, 6762)
Psychiatry visits	1776 (1723, 1847)	1850 (1739, 1943)	2019 (1938, 2120)	2355 (2214, 2449)	3378 (3175, 3581)
Occupational therapy visits	2335 (2265, 2428)	2420 (2323, 2589)	2640 (2561, 2746)	3079 (2987, 3264)	4150 (3901, 4441)
Palliative care visits	454 (440, 481)	477 (453, 496)	519 (498, 555)	606 (570, 648)	731 (702, 760)
Dietician visits	434 (421, 451)	456 (442, 488)	496 (481, 531)	579 (562, 614)	818 (777, 859)
Total Community Services Visits	11910 (11446, 12566)	12397 (11898, 13142)	13521 (13013, 14273)	15774 (15214, 16727)	22211 (21231, 23631)

**Table 4:** Average, 95% lower and upper CI results for the level of activity of PD doctors and nurses

Performance Indicator	Baseline	Scenario 1: Low use of CS	Scenario 2: Medium Use of CS	Scenario 3: High Use of CS	Scenario 4: Very High Use of CS
PD doctors service hours	8225 (7978, 8554)	7494 (7344, 7719)	7158 (6800, 7444)	6537 (6210, 6995)	5873 (5579, 6108)
PD nurses service hours	10357 (10150, 10978)	9854 (9460,10347)	9553 (8884, 10222)	8831 (8301, 9449)	8016 (7776, 8256)
PD doctors total FTEs	1.07 (0.995, 1.123)	0.97 (0.950,1.028)	0.93 (0.892, 0.985)	0.85 (0.824,0.884)	0.76 (0.722, 0.782)
PD nurses total FTEs	3.4 (3.230, 3.502)	3.2 (2.976, 3.36)	3.1 (2.883, 3.255)	2.9 (2.726,3.016)	2.6 (2.444, 2.756)
Total FTEs	4.47 (4.225, 4.625)	4.17 (3.926,4.388)	4.03 (3.775, 4.240)	3.75 (3.550,3.900)	3.36 (3.166, 3.538)
- *Treatment Costs*: The results regarding the expected hospital costs, community services costs, and total PD treatment costs are presented in Tables 5 and 6, and Fig. 6. These results show that hospital treatment costs should decline as policies regarding a higher use of community services are implemented. The rate of the decline becomes more important as higher fractions of patients are treated in community services. The hospital costs under the baseline scenario are £3,363,050 and these are expected to go down to £3,126,780 and £3,007,710 that is a percentage decrease of 7% and 11% for scenarios 1 and 2 respectively. With regard to scenarios 3 and 4, the decrease in the hospital costs is more important at £2,762,790 and 2,494,460, which is equivalent to a percentage decrease of 18% and 26%.The trend of the community services costs is similar to that of the number of visits to community services. As more patients are directed towards these services, their associated treatment costs go up by 4% to £755,434 under scenario 1, 14% to £824,044 under scenario 2, and 33% to £961,398 under scenarios 3 from £725,954 under the baseline scenario. This upward trend becomes substantially higher for scenario 4 as they reach £1,356,124, which is equivalent to an 86% increase compared to the baseline scenario.With regard to the total PD treatment costs, which includes both the hospital costs and the community services costs, adoption of policies to shift a higher number of patients to treatment in community services should lead to reduction in the total PD treatment costs. These saving are made possible as the decrease in hospital costs offset the increase in that of the community services leading to net gains with regard to total PD treatment costs. It is, however, interesting to notice that total PD treatment costs are on a steady decreasing pattern reaching their minimum under scenario 3. The pattern is then reversed when considering scenario 4. Compared to the baseline scenario, the percentage decrease in total PD treatment costs is 5%, 6.3%, and 9% under scenarios 1, 2, and 3 respectively. This percentage gain in total PD treatment costs is then reduced to only 5.8% under scenario 4 as these costs raise again to reach a level higher than that of scenarios 2 and 3 and close to that of scenario 1.

**Table 5:** Average, 95% lower and upper CI results for hospital costs

Performance Indicator	Baseline	Scenario 1: Low use of CS	Scenario 2: Medium Use of CS	Scenario 3: High Use of CS	Scenario 4: Very High Use of CS
PD doctors cost	1,809,500 (1,755,215; 1,881,880)	1,648,680 (1,549,759; 1,731,114)	1,574,760 (1,511,770; 1,669,246)	1,438,140 (1,366,233; 1,524,428)	1,292,060 (1,214,536; 1,343,742)
PD nurses cost	1,553,550 (1,506,944; 1,662,299)	1,478,100 (1,418,976; 1,566,786)	1,432,950 (1,389,962; 1,490,268)	1,324,650 (1,258,418; 1,377,636)	1,202,400 (1,142,280; 1,274,544)
Total Hospital Costs	3,363,050 (3,262,159, 3,544,179)	3,126,780 (2,968,735, 3,297,900)	3,007,710 (2,901,732, 3,159,514)	2,762,790 (2,624,651, 2,902,064)	2,494,460 (2,356,816, 2,618,286)

**Table 6:** Average, 95% lower and upper CI results for community services and total PD treatment costs

Performance Indicator	Baseline	Scenario 1: Low use of CS	Scenario 2: Medium Use of CS	Scenario 3: High Use of CS	Scenario 4: Very High Use of CS
Physiotherapy cost	135,052 (126,949, 140,454)	140,600 (133,570, 150,442)	153,368 (145,700; 159,503)	178,904 (171,748; 191,427)	256,690 (243,856; 272,091)
SLT cost	322,272 (306,158; 341,608)	335,424 (322,007; 348,841)	365,856 (354,880; 391,466)	426,912 (405,566; 456,796)	612,384 (581,765; 655,251)
Psychiatry cost	88,800 (85,248; 92,352)	92,500 (86,950; 97,125)	100,950 (96,912; 105,998)	117,750 (110,685; 123,638)	168,900 (158,766; 180,723)
Occupational therapy cost	135,430 (130,013; 144,910)	140,360 (136,149; 147,378)	153,120 (143,933; 162,307)	178,582 (171,439; 185,725)	240,700 (228,665; 250,328)
Palliative care cost	22,700 (21,792; 24,062)	23,850 (22,419; 25,520)	25,950 (24,393; 27,248)	30,300 (29,391; 32,421)	36,550 (35,088; 38,012)
Dietician cost	21,700 (20,832; 23,219)	22,700 (21,338; 23,835)	24,800 (23,808; 25,792)	28,950 (27,792; 30,108)	40,900 (38,855; 43,354)
Total community services Costs	725,954 (690,992, 766,605)	755,434 (722,433, 793,141)	824,044 (789,626, 872,314)	961,398 (916,621, 1,020,115)	1,356,124 (1,286,995, 1,439,759)
Total PD treatment Costs	4,089,004 (3,953,151, 4,310,784)	3,882,214 (3,691,168, 4,091,041)	3,831,754 (3,691,358, 4,031,828)	3,724,188 (3,541,272, 3,922,179)	3,850,584 (3,643,811, 4,058,045)

**Fig. 6:**
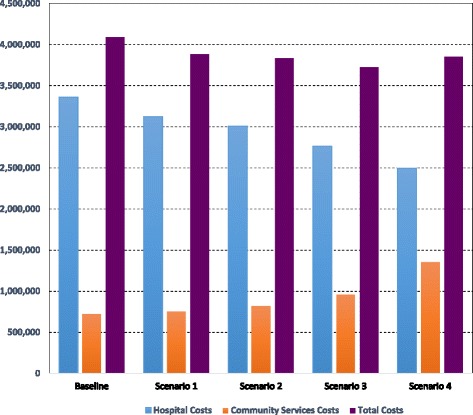
Expected hospital costs, community services costs, and total PD treatment costs

## Discussion

The study indicates that adoption of Community Services could lead to positive outcomes in terms of lower total PD treatment costs, decrease in the workload and activity levels of clinical staff in hospitals, and reduced requirements in terms of hospital resources and clinical headcount. This is helpful to policy makers who are required to provide treatment and high quality care for an increased number of PD patients in the context of reduced public funding for health. As more resources are freed in hospitals, this opens up the possibility to absorb the increasing demand for PD treatment and, consequently, reduce the time PD patients need to wait before commencement of treatment.

This positive impact within hospitals, however, must be viewed in conjunction with the increased costs and workload that shifting patients to community services brings. Successful implementation of any such policy involving shifting across sectors must be supported by careful processes of capacity management in community services, work-force planning and training programs. Revised procedures of commissioning and purchasing of services for PD patients must be in parallel with effective integration between health and social services. It is critical for policy makers to adopt a holistic approach to the management and treatment of PD patients. Focusing on the benefits of the policy on one element delivering the treatment (hospitals) and ignoring the spill over effects on the other element (community services) may lead to the opposite of what the policy is intended to achieve.

From a scale perspective, the overall performance gains, which could be achieved at the UK level could be important. The results reported in this study are for a single Trust only, but given that there are more than 350 NHS Trusts in the UK [8], the potential magnitude of total savings in PD treatment costs is significant. As the NHS is in financial deficit (£471 Million in the financial year 2014/15), the important potential cost reductions resulting from a wider use of Community Services can contribute toward much needed sustainable solutions.

The results also suggest that there is an “optimal” fraction of PD patients, which could be shifted to Community Services. The overall PD treatment costs have a decreasing pattern up to the scenario where the shift is at a level of 40%; but total costs increase beyond this percentage. This finding has important implications with regard to scaling of Community Services and the best allocation of patients between the hospital and Community Services settings.

In addition to the benefits from the care delivery perspective, there is strong evidence that patients find community services a friendlier environment and where staff are more able to deliver tailored care accounting for personal needs and circumstances of patients [6]. This “human” aspect has been highlighted by the advocates of a wider use of these services as positive psychological impact of treatment in community services is as important as the medical treatment itself. This is especially important for those patients facing social exclusion, which can exacerbate some PD symptoms (such as depression and dementia). As such, community services can play an important role in reversing PD patients’ low satisfaction with quality of care and their complaints about the lack of attention towards their psychological welfare.

The importance of community services in the treatment and management of patients with PD was highlighted in policy documents and treatment delivery framework about a decade ago [5, 38]. However, the acknowledgment of their important role gained momentum in recent years due to increased demand and the pressures to deliver efficient and high quality care to PD patients [39, 40]. The results of this research are in line with this renewed policy direction and add to the evidence that Community Services can be an important element of an innovative PD care delivery model. Making such a model successful, however, requires an alignment between capacity and resources such as PD nurses and therapists and the level of demand for care in community services. In this context, the processes of workforce planning and training programs need to be designed with current and future shifts toward community services [40, 41].

Adequate resource availability is important to ensure quality of the care provided in community services to maximise impact on quality of life (5,42,43]. The integration between health services and social services is another key driver of quality of care in Community Services [39, 42]. As care in community services is organised in Multidisciplinary Teams (MDT) including PD nurses, therapists, and social services workers, it is critical that the mix of resources to constitute the MDT are available and that the MDT functions in a coherent way to meet the multiple needs of PD patients. This requires high levels of coordination between health and social services in terms of the management of care plans for patients, provision of services in a timely manner, and the planning of workforce development and training activities. PD nurses fulfil a pivotal role as part of the MDT and in promoting up take of community services at periodical meetings with patients where treatment needs and care plans are discussed [39, 43].

To ensure equity of access, health service commissioners would need to review disparities in community provision between urban and rural areas across the UK.

The findings of the research are based on results obtained from the application of the DES model to a single (albeit large) Trust in the UK. It is expected that the general findings regarding the positive impact of increased use of community services may be generalised to other Trusts of various sizes in the UK. The model developed reflects the patients’ pathways recommended by policy documents in the UK such as NICE guidelines [5] and which were confirmed to be relevant and practicable by PDNSA nurses in the Trust. Cost data were from the Department of Health and not expected to vary widely in different Trusts. However, it is possible that the “optimal” fraction of patients to be shifted to community services (40% in the Trust studied in the current research) is different in other Trusts due to varying patient numbers and distribution of patients between the four stages of the disease. The methodology and model is easily transferable to other contexts, as variables are explicit and variation can be accounted for, generating new optimal service configuration. Where Trusts operate a significantly different patient pathway and/or community services are unable to provide the full range of services, the current model would require significant revision.

The model was developed with the active engagement of policy makers and staff involved in the provision of PD treatment and care. This collaborative effort led to a feeling of problem ownership by the stakeholders and was a critical factor in the adoption of the model and its results. This positive outcome was also facilitated by developments in user friendly Simulation software, lowering the technical barriers faced by health managers and enabling them to play a more assertive role in building and using simulation models. The current study reflects well this evolutionary process and the move from the times when model building was the sole responsibility of technical experts to the current contexts where this responsibility is shared by the problem owners and the modellers [44].

The current study also highlights the growing role Simulation modelling is playing in the context of the shift towards more “evidence based” decision making in the health sector. It has been observed that in several instances, health managers had a clear idea about the decisions to be made to improve performance, but lacked the evidence to make a case to decision makers. By providing that evidence, simulation modelling fills this gap and allows policies to be selected and implemented with confidence and with a clear vision about what is expected in the future.

The study, of course, has some limitations. The model represented a single Trust and this has implications for generalizability of the results as discussed earlier. The model did not take account of the psychological attributes of the patients and how these could influence the progression of the disease and the effectiveness of the treatment. Furthermore, as it is virtually impossible to wholly represent real world complexity of health systems [23], the results of this study should be interpreted with care. The model includes a number of assumptions and simplifications, and increased complexity would be introduced by: the different age categories of patients; co-existence of other medical conditions in addition to PD for some patients; socio-economic status of patients; effects of syndromes such as lack of mobility, depression, and dementia on attendance of planned appointments in hospitals and community services. Opportunity costs to patients and indeed carers are important but outside the scope of this current study. Future research and model development which integrates both the provider and patients’ perspectives are needed.

## Conclusions

The number of patients affected by PD is increasing and this trend is expected to continue and magnify in line with the UK population structure and ageing. This creates significant pressures on PD and wider services in the absence of a cure. Current evidence suggests that treatment of degenerative neurological diseases such as PD is expensive, inefficient, inaccessible to a considerable number of patients, and of sub-optimal quality. Financial austerity and cuts to public funds allocated to the health sector demand innovative solutions for care delivery leading to efficiencies across a healthcare economy. The current study is particularly timely against this background and explores the possibility and scale of efficiency gains, which could be achieved if Community Services are more widely adopted and become an integrated part of the PD treatment process.

In terms of policy, current PD treatment and patient management guidelines and procedures are a decade old, and may no longer be appropriate in the current NHS environment. The formal evaluation of innovative treatment procedures such as the use of community services could offer policy makers insights about more effective ways of caring for PD patients whilst enhancing quality. The advantages of community services need to be effectively shared with PD patients and carers to help inform management choices and care plans.

The successful application of Simulation Modelling in this research and the enthusiasm and engagement of key participants strengthens the argument for co-developing policy improvement. As the complexity of health systems increases in an environment of constrained resources, so too does the need for evidence based decision making. Simulation modelling is well positioned to provide that evidence and allow policy makers to face upcoming challenges with greater confidence.

## Additional file

Additional file 1: BMC HSR Data for simulation model. **Table S7.** Data used for the simulation model. Table providing details of parameters used in the simulation model including the source, distribution type and the value entered in the model. (DOCX 14 kb)

## Abbreviations

CI: Confidence interval
DES: Discrete event simulation
GPs: General practitioners
NHS: National health service
NHS: National health service
NICE: National institute for health and care excellence
PD: Parkinson’s disease
SLT: Speech and language therapy Uk United Kingdom
