# Supplementary material for: A discrete event simulation model to evaluate the use of community services in the treatment of patients with Parkinson’s disease in the United Kingdom
Source: BMC Health Serv Res. 2017 Jan 18;17:50. doi: 10.1186/s12913-017-1994-9 (PMC5241966; doi:10.1186/s12913-017-1994-9)
Supplement: Additional file 1: — BMC HSR Data for simulation model. Table S7. Data used for the simulation model. Table providing details of parameters used in the simulation model including the source, distribution type and the value entered in the model. (DOCX 14 kb) [file 12913_2017_1994_MOESM1_ESM.docx]

Table 7. Data used for the simulation model

| **Parameter** | **Source** | **Distribution Type** | **Value entered in the model** |
| --- | --- | --- | --- |
| **Demand** | | |  |
| Existing patient arrival | HES data or user specified | Poisson | Existing patient size = 1000 |
| Percentage of patients falling into each category, i.e., diagnosis, maintenance, complex and palliative. | User specified | Multinomial | Diagnosis = 10%, Maintenance = 60%, Complex = 20% and Palliative = 10% |
| Yearly increase in new patients | User specified | Multinomial | 5% |
| Percentage of suspected PD patients | User specified | Bernoulli | 90% |
| Percentage of patients presenting through General Practitioner | HES data or user specified | Multinomial | 75% |
| Percentage of patients presenting through A&E | HES data or user specified | Multinomial | 15% |
| Percentage of patients presenting through Outpatients | HES data or user specified | Multinomial | 5% |
| Percentage of patients presenting through other hospital department, e.g. care of the elderly | HES data or user specified | Multinomial | 5% |
| **Treatment pathway (in hospital)** | | |  |
| Time between initial outpatient screening to a specialist (i.e. neurologist) for first PD diagnosis | User specified | Uniform | [2, 4] weeks |
| Time between first diagnosis to the start of treatment | User specified | Uniform | [1,4] weeks |
| If PD is suspected, what percentage of patients is actually diagnosed for PD? | User specified | Bernoulli | 90% |
| The number of times (in a given year) each patient is seen by a Neurologist (by patient type). | User specified | Fixed | Diagnosis = 1, Maintenance = 2, Complex = 3 and Palliative = 4. |
| The number of times (in a given year) each patient is seen by a specialist PD nurse (by patient type). | User specified | Fixed | Diagnosis = 2, Maintenance = 3, Complex = 4 and Palliative = 6. |
| The time it takes for a neurologist to treat patients (in minutes) | User specified | Mean | 60 minutes |
| The time it takes for a specialist PD nurse to treat patients (in minutes) | User specified | Mean | 60 minutes |
| **Community services pathway** | | |  |
| Percentage of patients referred to community services, i.e., physiotherapy, psychiatry, speech and language therapy (SLT), occupational therapy (OT), palliative care, dietician. | User specified | Multinomial | Physiotherapy = 45%, psychiatry = 22.5%, SLT = 42.5%, OT = 35%, Palliative = 7.5%, Dietician = 7.5% |
| The number of times (in a given year) patients are referred to physiotherapy (by patient type). | User specified | Poisson | Diagnosis = 1  Maintenance = 2  Complex = 2  Palliative care = 3 |
| The number of times (in a given year) patients are referred to psychiatry (by patient type). | User specified | Poisson | Diagnosis = 1  Maintenance = 2  Complex = 2  Palliative care = 3 |
| The number of times (in a given year) patients are referred to speech and language therapy (by patient type). | User specified | Poisson | Diagnosis = 1  Maintenance = 2  Complex = 2  Palliative care = 3 |
| The number of times (in a given year) patients are referred to occupational therapy (by patient type). | User specified | Poisson | Diagnosis = 1  Maintenance = 2  Complex = 0  Palliative care = 0 |
| The number of times (in a given year) patients are referred to palliative care (by patient type). | User specified | Poisson | Diagnosis = 0  Maintenance = 0  Complex = 2  Palliative care = 4 |
| The number of times (in a given year) patients are referred to a dietician (by patient type). | User specified | Poisson | Diagnosis = 1  Maintenance = 1  Complex = 2  Palliative care = 3 |
| **Disease Progression** | | |  |
| Diagnosis to Maintenance | User specified | Triangular distribution | [min = 1 year, average = 2 years, maximum = 4 years] |
| Maintenance to Complex | User specified | Triangular distribution | [min = 2 year, average = 3 years, maximum = 4 years] |
| Complex to Palliative | User specified | Triangular distribution | [min = 3 year, average = 5 years, maximum = 7 years] |
| Palliative to Death | User specified | Triangular distribution | [min = 3 months, average = 6 months, maximum = 1 year] |
| **Cost** |  |  |  |
| A&E attendance including hospital admissions | HRG codes (reference costs) | Mean | £2,233 |
| Neurologist | HRG codes (reference costs) | Mean | £220 |
| Imaging | HRG codes (reference costs) | Mean | £100 |
| Unit cost for PD specialist nurse | User specified | Mean | £150 |
| Physiotherapy | HRG codes (reference costs) | Mean | £38 |
| Psychiatry | HRG codes (reference costs) | Mean | £50 |
| Occupational therapy | HRG codes (reference costs) | Mean | £58 |
| Speech and language therapy | HRG codes (reference costs) | Mean | £96 |
| Palliative care | HRG codes (reference costs) | Mean | £50 |
| **Salary** | | |  |
| PD Nurse | User specified | Mean | £36,303 |
| PD Doctor | User specified | Mean | £80,810 |
| **Number of resources** | | |  |
| PD nurse | User specified | Fixed | 5 |
| PD Doctors | User specified | Fixed | 2 |
